# Engineering Multiscale Vasculature: Biological Principles, Design Constraints, and Advanced Biofabrication Strategies for Functional Vascular Networks

**DOI:** 10.3390/biomimetics11090668

**Published:** 2026-09-17

**Authors:** Behnaz Eftekhari, Shawn P. Grogan, Darryl D. D’Lima

**Affiliations:** Scripps Health, Shiley Center for Orthopaedic Research and Education at Scripps Clinic, 10666 North Torrey Pines Road, La Jolla, CA 92037, USA; eftekhari.behnaz@scrippshealth.org (B.E.); grogan.shawn@scrippshealth.org (S.P.G.)

**Keywords:** biofabrication, multiscale vascular engineering, perfusable vascular networks

## Abstract

Creating functional multiscale vascular networks remains a major challenge in tissue engineering and regenerative medicine. Native vasculature spans a wide range of diameters, from large elastic arteries to microscale capillaries, supporting vital functions such as perfusion, barrier regulation, mechanotransduction, and immune surveillance. Replicating this complexity requires biomaterials and fabrication techniques that enable hierarchical branching, preserve endothelial and mural cell phenotypes, and withstand physiological hemodynamic forces. We review biophysical design constraints, including diffusion limits, shear stress, mechanical compliance, and endothelial specialization, in the context of vascular developmental biology to outline a multiscale engineering framework for vascular graft design. Several promising scaffold systems are evaluated including electrospun fibers, decellularized matrices, cell-sheet constructs, and 3D bioprinting methods such as sacrificial-template fabrication, based on their respective advantages, limitations, and translational progress. Microvascular and organ-on-a-chip models are evaluated as high-fidelity platforms for studying human vascular physiology and disease. Finally, we discuss cellular sources for vascularization, including primary endothelial cells and iPSC-derived vascular lineages, as well as coculture and preconditioning strategies to enhance vessel maturation. While previous reports have reviewed individual vascular biofabrication methods, we integrate vascular biology, biophysical design constraints, fabrication strategies, and cell sources across macrovascular and microvascular scales. A multiscale approach is critical for selecting and combining approaches to meet vessel-specific requirements. Collectively, these advances may facilitate the development of perfusable, stable, and tissue-specific vascular networks and support the translation of multiscale vascular grafts toward clinical applications.

## 1. Introduction

The development of a functional vascular supply is essential for the survival, maturation, and integration of engineered tissues. In vivo, the vascular system is a complex, branched network that spans tens of thousands of miles, providing continuous delivery of oxygen, nutrients, and hormones, while removing metabolic waste [1]. Vessel diameters vary significantly, ranging from large elastic arteries measuring several centimeters to capillaries as small as 10–15 µm, reflecting highly specialized structural and functional adaptations matched to the metabolic demands of different organs [2]. This multiscale hierarchy is formed during embryogenesis via vasculogenesis and angiogenesis and sustained throughout life through coordinated interactions between endothelial cells (EC), mural cells (smooth muscle and pericytes), the extracellular matrix, and hemodynamic forces [1].

Replicating this complexity through engineering remains a significant challenge. Engineered vessels must replicate the physical structure (lumen diameter, branching pattern, and wall composition), as well as dynamic features such as shear responsiveness, barrier regulation, vasomotor tone, and immunological compatibility [3]. Biological requirements are important for successful integration with host tissues and for preventing thrombosis, hypoxia, and maladaptive remodeling. Because the limits of diffusion restrict oxygen transport to 150–200 µm from a perfused vessel, microvascularization is necessary for maintaining viability in tissues or cellular constructs thicker than a few hundred micrometers [4,5].

To meet these needs, several biomaterials and biofabrication processes have been developed. Electrospinning can generate nanofibrous scaffolds that emulate native extracellular matrix [6,7], while decellularized matrices retain the biochemical and mechanical cues of native vessels [8,9]. Cell-sheet engineering creates dense ECM-rich tissue with inherent vascular potential, and advanced 3D-printing solutions can manipulate vascular geometry across various length scales [10]. Microvascular organ-on-a-chip systems serve as high-fidelity platforms for studying vascular physiology, disease, and drug responses [11]. Meanwhile, the range of cell sources available for vascularization is expanding beyond conventional endothelial cells, with endothelial progenitor and iPSC-derived endothelial and mural lineages providing scalable patient-specific sources. Cell culture techniques, stromal enhancement, and biomechanical preconditioning further aid vessel maturation and stability [12].

Previous reviews covered vascular tissue engineering, biomaterials, and individual biofabrication approaches. However, not enough attention has been given to selecting and combining these strategies to meet the diverse biological and biomechanical requirements of different vascular scales. In this review, we survey vascular developmental biology, engineering constraints, fabrication strategies, and cellular sources within a multiscale framework. We consider how factors such as diffusion limits, hemodynamic forces, endothelial specialization, and host integration influence the selection and design of approaches for macrovascular and microvascular engineering. We evaluate recent advances in electrospinning, decellularized matrices, cell-sheet engineering, 3D bioprinting, sacrificial-template fabrication, and microvascular organ-on-a-chip systems for potential to meet these requirements. By coupling biological considerations with engineering strategies across different vascular scales, this review establishes a useful framework for developing clinically suitable, perfusable, and durable vascular networks.

## 2. Physiology and Development of the Vascular Network

The highly complex vascular network functions as the animal’s central transport system, supporting tissues throughout an extensive circulatory system, extending across tens of trillions of cells [13,14]. This system is surprisingly large, spanning a range of vessel sizes, from the large aorta (a few centimeters in width) down to capillaries with luminal diameters of approximately 10–15 µm [13,14,15]. This wide variability supports specialized structural, metabolic, and biomechanical functions of diverse tissues and organs. Replicating this hierarchical organization is one of the most challenging tasks in the field of tissue engineering and regenerative medicine [1]. This is largely because achieving long-term vascular viability and function requires replicating the macro- and microvascular branching architecture integrated with a functional endothelium, appropriate biomechanical properties, and biological and biomechanical signaling [16].

**Figure 1:**
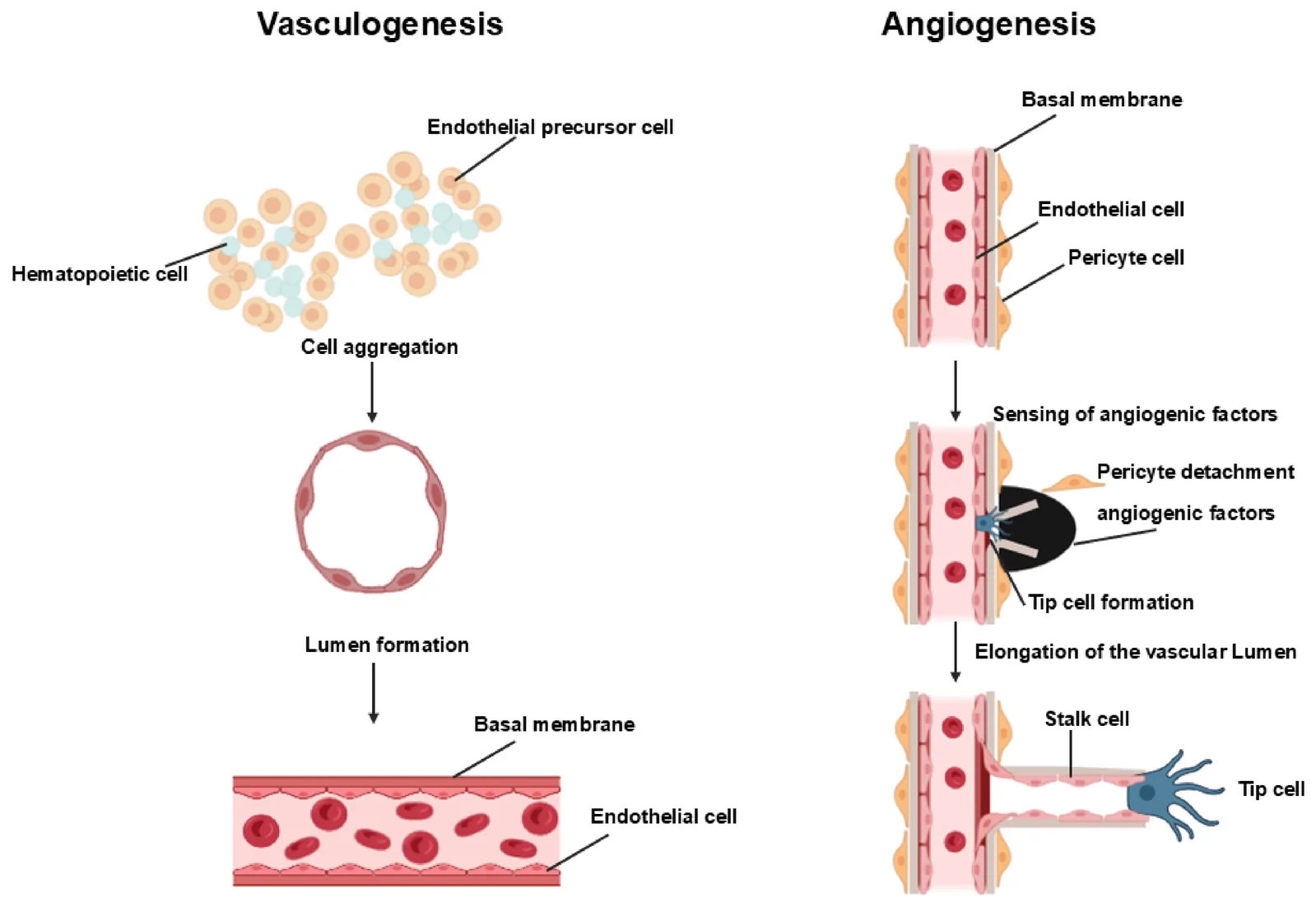
Vascular development. Vasculogenesis (**left**) is the de novo formation of vessels as mesoderm-derived angioblasts aggregate to form blood islands that fuse into a primitive vascular plexus. Angiogenesis (**right**) widens and reconfigures this plexus, allowing endothelial cells to invade the surrounding matrix, peripheral pericytes to detach, and migratory tip cells to evolve toward pro-angiogenic cues while proliferating stalk cells follow. These processes coordinate to construct a hierarchical vascular network.

The native vascular network forms during embryogenesis via two processes, vasculogenesis and angiogenesis (Figure 1). In vasculogenesis, mesoderm-derived angioblasts aggregate as blood islands forming the primitive vascular plexus, which establishes the initial pathways to embryonic blood flow. During angiogenesis, the plexus is reshaped and extended by endothelial cells proliferating, migrating, and invading the extracellular matrix giving rise to sprouts with lumens that elongate and form functional networks. The endothelial specification, branching morphogenesis, and arteriovenous identity is tightly regulated by VEGF–Notch, angiopoietin–Tie2, and ephrinB2–EphB4 signaling pathways [17,18,19].

Endothelial cells (ECs) form the innermost lining of all blood vessels but rely upon supporting mural cells for stability and maintenance of their function. Pericytes (which reside on capillary and post-capillary venules) mediate deposition of basement membranes, paracrine signal transduction, regulation of endothelial barrier integrity and microvascular hemodynamic stability. In larger vessels, vascular smooth muscle cells (VSMCs) and fibroblasts organize into concentric layers, where muscle strength is conferred in relation to tensile strength, contractility, and elasticity. The interaction between ECs and mural cells is crucial for vessel patency, permeability regulation, and hemodynamic adaptation, and its disruption underlies vascular pathologies, such as diabetic microangiopathy and atherosclerosis [20,21,22].

The functional activity of blood vessels is related to their size and structural composition [16]. Large elastic arteries, such as the aorta, accommodate the pulsatile output of the heart through elastin-rich lamellae and several layers of VSMCs. Medium-sized muscular arteries channel blood flow into targeted tissue beds and regulate vascular resistance, while arterioles (≈10–1000 µm in diameter) act as the chief regulators of blood pressure and local perfusion through finely controlled vasoconstriction and vasodilation [16]. The capillaries at the terminal tips of the vascular tree have a single layer of endothelial cells, resting on a basal lamina closely associated with pericytes, thereby maximizing surface area for gas, nutrient, and waste exchange. This structural hierarchy dictates different engineering requirements: large-vessel grafts should achieve sufficient burst pressure and appropriate compliance; microvascular constructs must feature high-resolution patterning, a controlled permeability, and strong endothelial–pericyte interactions [3,23].

While the vascular tree provides a blueprint, a developing and engineering artificial vasculature is informed by developmental biology and physiology. To achieve functional translation the following critical features have to be integrated in an engineering platform: (i) branching to represent the hierarchical distribution of blood flow over multiple scales, (ii) incorporation of mural support cells to increase vessel stability and support adaptive remodeling, (iii) organ-specific endothelial phenotypes for efficient barrier formation and exchange, and (iv) mechanical and biochemical compatibility within the host vasculature for rapid anastomosis. Advances in stem cell technologies, biofabrication, and biomaterials must converge to recapitulate these complex biological properties, thereby advancing engineered vasculature from the bench to the clinical setting [17,24].

## 3. Biological Considerations for Vascular Engineering

Engineering vascular networks are constrained by interdependent requirements for mass transport, hemodynamic function, endothelial stability, and host integration [2,16]. Unlike synthetic tubing or microfluidic systems, engineered vasculature must simultaneously maintain structural integrity, tissue-appropriate function, and capacity for remodeling [3,25]. Failure to meet these requirements can result in thrombosis, tissue hypoxia, or poor integration with the host vasculature [16,26,27].

The most fundamental limitation is the diffusion-limited transport of oxygen and metabolites. Cells can only survive within 150–200 µm of a perfused vessel. Above this distance, cells develop hypoxia, acidosis, and finally necrosis due to insufficient nutrient delivery and waste clearance [1,16,25]. As a result, engineered tissue constructs thicker than a few hundred micrometers must incorporate organized microvascular networks or perfusable channels. Physiologically relevant vessel spacing and branching density, therefore, are a basic design necessity for tissue viability.

**Figure 2:**
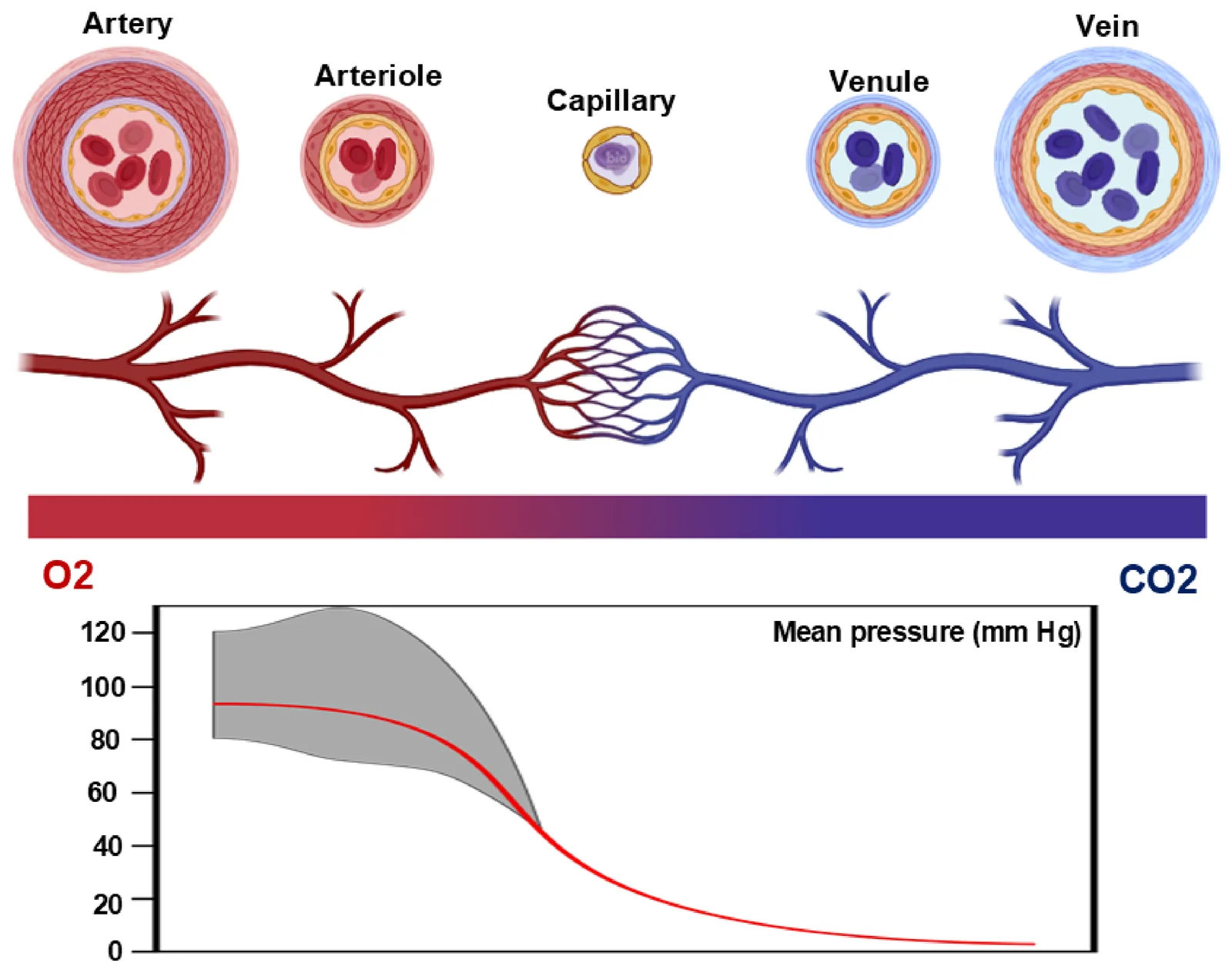
Schematic illustration of multiscale vascular organization and hemodynamic gradients across the vascular tree. The diagram depicts the transition from large elastic arteries to veins, highlighting progressive changes in vessel diameter, wall structure, and pulsatile pressure profiles that govern blood flow, mechanical loading, and vascular function.

Engineered vascular systems must also withstand the physiological mechanical environment of blood flow. Large arteries experience significant pulsatile pressures, resulting in large dynamic cyclic strain, while capillaries encounter much lower pressures but are still dependent on fine barrier regulation (Figure 2) [2]. Normal shear stresses induced by blood flow in veins range between 1 and 6 dyn/cm^2^, and in arteries between 10 and 20 dyn/cm^2^, and are essential for controlling endothelial cell alignment (and its accompanying nitric oxide and anti-thrombogenic activity). Failure to reproduce these hemodynamic conditions leads to endothelial dysfunction and a prothrombotic phenotype that is not readily reproduced in vitro. As a result, engineered vascular channels must have luminal surfaces and geometries that protect their structural integrity under cyclical loading and prevent rupture or stenosis [26,27].

A non-thrombogenic endothelium is essential for perfused constructs. Polymeric or hydrogel media readily promote platelet adhesion and thrombus formation in the absence of an endothelial lining [28]. Endothelialization provides selective permeability for communication and nutrient exchange, and secretes antithrombotic factors, for example, prostacyclin and nitric oxide [24,29]. In addition, EC origin significantly influences long-term function: arterial, venous, and microvascular ECs display distinct, bed-specific phenotypes that shape long-term function and compatibility with the local hemodynamic environment. ECs exhibit specific gene expression profiles, barrier type and properties, and flow responses. Therefore, precisely reproducing an appropriate endothelial phenotype is critical when designing and engineering vascular constructs that mimic the properties of a normal vascular bed [30,31].

Engineered tissues need to rapidly integrate with the host vasculature to ensure efficient perfusion after implantation as delayed vascular integration can lead to ischemia and graft failure. This incorporation can be achieved via surgical anastomosis, in which the lumens of host and graft vessels are directly connected, or by inosculation, whereby preformed microvessels within the implanted construct spontaneously fuse with ingrowing host microvessels [32]. Inosculation rates and efficiency depend on important factors such as vessel density, existing lumen patency, and pro-angiogenic signals. Thus, biomaterial design should consider biochemical cues in addition to structural fidelity to accelerate endothelial sprouting, mural cell recruitment, and vascular remodeling [24,33].

Another major consideration is the extensive diversity of vascular beds in different organs. Microvessels in the brain establish a blood–brain barrier, with tight intercellular junctions that block transcytosis, whereas liver sinusoids possess a fenestrated endothelium that facilitates optimal macromolecular exchange [22,34]. Recapitulation of context-dependent microvascular properties requires careful selection of biomaterials, extracellular matrix components, and biochemical cues for endothelial specialization. These attributes are necessary for engineered vessels to reproduce physiological transport dynamics without pathological permeability [35,36].

Lastly, engineered vascular constructs should facilitate longer term remodeling without eliciting an immune reaction. Non-biological surfaces or incompletely endothelialized grafts can stimulate complement activation and inflammatory cascades that precipitate thrombosis or graft failure [37,38]. Engineered vessels must maintain the structural ability to remodel in response to growth, mechanical stress, and injury, to mirror the adaptive plasticity of native vasculature [39]. The balance between living cells and biodegradable scaffolds, which can be progressively degraded and replaced by host cells, is necessary for localized deposition of cell-derived extracellular matrix at the site of action [7,40]. These critical requirements limit the biological design space for successful vascular tissue engineering.

Satisfying conflicting biological requirements within a single tissue-engineered vascular construct remains challenging. For example, increasing scaffold strength tends to reduce porosity which impacts cell infiltration and nutrient transport. On the other hand, increasing porosity or biodegradability may jeopardize stability under physiological flow. Stable endothelialization and rapid inosculation must be achieved without compromising patency or tissue-specific endothelial function. Because these trade-offs differ significantly across vessel scales, a single design approach is unlikely to be appropriate for both large vessels and microvascular networks. Therefore, vascular construct design requires a coordinated optimization of mechanical properties, mass transport, and cellular function.

Together, these considerations define the complex biological design space for functional vascular constructs. Meeting these requirements simultaneously remains a major challenge and requires coordinated integration of multidisciplinary and multiscale strategies [18,41,42,43,44,45,46].

## 4. Macro and Microvascular Graft Design

The clinical demand for vascular grafts ranges from large-caliber aortic replacements to small-diameter coronary or peripheral vessel bypass conduits. Autologous vascular grafts remain the treatment of choice, but their scarcity has prompted considerable efforts to develop engineered alternatives [47]. Physiological and mechanical requirements for large (>6 mm) versus small (<6 mm) diameter grafts are notably different, making design and material selection dependent on graft size. Conduits employed in high-flow vessels (e.g., large-caliber grafts implanted into the aorta or femoral artery) must withstand significant hemodynamic loads, including pulsatile pressure, cyclic strain, and elevated shear stress. Synthetic polymers, including Dacron (polyethylene terephthalate) and expanded polytetrafluoroethylene (ePTFE), have been widely used in clinical applications in high-flow vessels with durable, long-term patency, structural robustness, and stability under arterial pressures, and they are now well-established in the market for such applications [47].

However, non-living grafts cannot remodel or grow with the host and remain susceptible to infection and chronic inflammation. This limitation is even more crucial in a pediatric setting, where the grafts need to remodel and support somatic growth over time. Biodegradable, cellularized, large-caliber grafts have been developed to overcome this challenge. These constructs are commonly synthesized from polyglycolide (PGA) or polycaprolactone (PCL) polymers seeded with autologous vascular cells, and have shown promising results in preclinical and early clinical studies, with enhanced growth and host integration compared to generic synthetic grafts [47,48].

Engineering conduits for coronary or peripheral artery bypass has been considerably more difficult [49]. Materials well-suited for large-caliber use often do not perform well at smaller diameters, as the risk of thrombus formation, neointimal hyperplasia, and flow dysfunction is increased in low-shear settings. Therefore, autologous saphenous vein and internal mammary artery grafts remain the clinical gold standard [47]. To overcome these challenges, several innovative strategies are discussed next.

### 4.1. Electrospun Scaffolds

Electrospinning is one of the most versatile and practical method for manufacturing scaffolds that mimic the fibrous structure of natural ECM [6]. Electrospinning allows very fine control of fiber alignment, diameter, and porosity, which are critical factors governing mechanical strength, cell adhesion, and nutrient diffusion. Due to these characteristics, electrospun scaffolds have attracted considerable interest in developing small- and medium-sized tissue-engineered vascular grafts [7].

Many synthetic and natural polymers have been electrospun to optimize trade-offs between performance and biocompatibility (Table 1). In general, natural polymers such as collagen, gelatin, elastin, and chitosan possess high bioactivity and support cell adhesion [41]. Synthetic polymers such as poly ε-caprolactone (PCL), poly lactic-co-glycolic acid (PLGA), polyester urethane urea (PEUU), and poly L-lactic acid-co-caprolactone (PLLA-CL) exhibit tunable mechanical properties and durability. Synthetic scaffolds can also support cellular infiltration and promote endothelial differentiation [6,7,42,43].

Electrospun grafts have demonstrated vascularization, patency, and remodeling in preclinical studies. Collagen-coated PLLA-CL scaffolds resulted in grafts with aligned endothelium that were patent for several weeks after implantation in rabbits [44]. Conduits constructed by electrospinning recombinant human tropoelastin/PCL-enhanced endothelialization, reduced platelet adhesion, and achieved mechanical behavior similar to that of native arteries [45]. In rats, phospholipid-coated PEUU aortic grafts maintained patency with low thrombosis for 24 weeks [46], while electrospun PCL aortic grafts remained patent up to 18 months [42]. These studies collectively emphasize the potential of electrospun scaffolds to support physiological flow and stimulate ECM remodeling, all of which are necessary components of functional vascular integration.

Composite scaffolds have also been developed to more closely mimic the tri-layered structure of native vessels by integrating hybrid polymers or multilayered structures, with porous layers for cell penetration and denser layers for burst resistance [50]. Examples include bilayer PCL/collagen scaffolds with graded porosity that promote smooth muscle cell (SMC) infiltration in the outermost layer and endothelial cell adhesion to the lumen [51], and reinforced electrospun PCL tubes incorporating a helical coil [52].

Despite promising advancements in electrospinning, several limitations remain. Dense fiber packing inhibits cellular infiltration and nutrient diffusion, and insufficient or incomplete remodeling and immune compatibility pose significant issues for translation. To improve porosity and hemocompatibility, several approaches, including salt or cryogenic leaching, sacrificial fiber removal, and surface functionalization with peptides or ECM proteins, have been applied [6,7,53]. Co-electrospraying cells or bioactive factors during fiber deposition offers potential for spatially controlling cell distribution and enhancing bioactivity [54].

In general, clinically successful translation will require a combination of scaffolds that provide biomechanical stability and support remodeling within physiologically compatible architectures.

### 4.2. Decellularized Matrices

Existing synthetic constructs lack the natural ultrastructure and matrix-bound signaling cues necessary for vascular cell function. ECMs obtained by decellularizing native vessels have been repurposed as highly promising scaffolds in vascular tissue engineering because of their biochemical composition and hierarchical structure. Decellularized scaffolds retain native collagen, elastin, glycosaminoglycans, and embedded cytokines, thus facilitating cellular adhesion, migration, and supporting vascular cell phenotype [8,9]. This capacity to recapitulate the mechanical anisotropy and bioactive milieu characteristic of native arteries makes them valuable for the development of small-caliber vascular grafts [55].

Several decellularization strategies have been proposed to achieve a balance between cellular removal and structural protection. These include physical, chemical, and osmotic treatments that minimize ECM degradation [56]. Crosslinking of ECM minimizes immunogenicity and increases durability with resistance to enzymatic degradation and calcification [57,58]. Dynamic conditioning in bioreactors induces matrix remodeling through ECM alignment, improves stiffness, and increases endothelial layer formation [58,59].

Hybrid scaffolds are being explored that combine decellularized ECM with synthetic polymers or bioactive molecules for synergistic activity [60]. Combining poly (1,8-octanediol citrate) with decellularized rat aorta decreased platelet adhesion and promoted vascular endothelial and smooth muscle cell attachment in vitro [61]. Hybrid scaffolds overcome the intrinsic mechanical fragility of the endogenous ECM while maintaining its biological signals. Electrospun PCL nanofibers superimposed on decellularized aortas increased mechanical properties and localized delivery of rapamycin alleviating SMC hyperproliferation and neointimal thickening [62]. Heparin-linked decellularized placental scaffolds have shown high anti-thrombogenicity, macrophage compatibility, and nearly 100% patency in rat aortic implants over one month [63,64]. High hydrostatic pressure decellularization of porcine radial arteries achieved full endothelial coverage and 100% patency in rat models within 4 weeks [65]. UV-crosslinked decellularized rabbit aortas implanted for several weeks exhibited collagen regeneration and smooth muscle differentiation, with lumen patency and mechanical compliance similar to native arteries [66].

The dynamic bioreactor conditioning is another determinant of matrix remodeling [67,68]. Perfusion culture of decellularized scaffolds maintained in a state of dynamic flow improves ECM alignment, increase stiffness, and enhance endothelial layer formation, thereby better recapitulating native vessel physiology. Finally, these observations indicate that mechanical pre-conditioning and chemical surface modification synergistically promote the integration and survival of decellularized vascular grafts [64].

The incorporation of growth-factor-binding domains, development of ECM-mimetic hydrogels, and inclusion of stem cell recruitment peptides in decellularized constructs has the potential for clinical application as bioactive, off-the-shelf grafts for self-endothelialization and adaptive remodeling [55,57,69]. However, variability in decellularization efficiency and damage to the ECM from sterilization remain important concerns. Residual DNA, collagen/elastin integrity, and retention of biomechanical properties are significant variables for long-term reproducibility [58,63,70]. Although greater than 90% short-term patency rates are often reported in small animals, longer-term remodeling and immune compatibility require validation in large-animal preclinical studies [55,63,65].

### 4.3. Cell-Sheet Engineering

In contrast with natural and synthetic scaffolds, cell-sheet engineering (CSE) is a scaffold-free method for synthesizing dense, ECM-rich tissue layers that preserve intercellular junctions and native adhesion proteins. Initially proposed by Okano and colleagues, a thermoresponsive substrate, such as poly(N-isopropylacrylamide) (PIPAAm), facilitates the attachment and detachment of confluent monolayers of adherent cells [71,72]. Traditional enzymatic passaging breaks down cell–cell adhesion molecules along with the ECM. CSE preserves the native microenvironment supporting crosstalk between cells and their ECM which is especially important for angiogenic potential [73].

Cell sheets formed from densely contiguous layers of endothelial or stromal cells can be stacked or wrapped in series of nested layers to form multicellular constructs without the disadvantages of artificial scaffolding. Preserving cell–cell junctions and matrix proteins facilitate inosculation and capillary formation after implantation. Stacked endothelial cell-containing and stromal/smooth muscle cell sheets have been shown to generate prevascularized constructs that promote capillary network formation, host vascular integration, and functional tissue perfusion after transplantation [74,75]. Dual-layer strategies in which an endothelialized sheet is wrapped around an osteogenic or smooth muscle sheet provide prevascularized grafts with improved perfusion and remodeling [76]. CSE may be useful for constructing thick functional metabolically active grafts in conditions that may impair diffusion-limited hypoxia, such cardiac and hepatic tissues [77].

**Figure 3:**
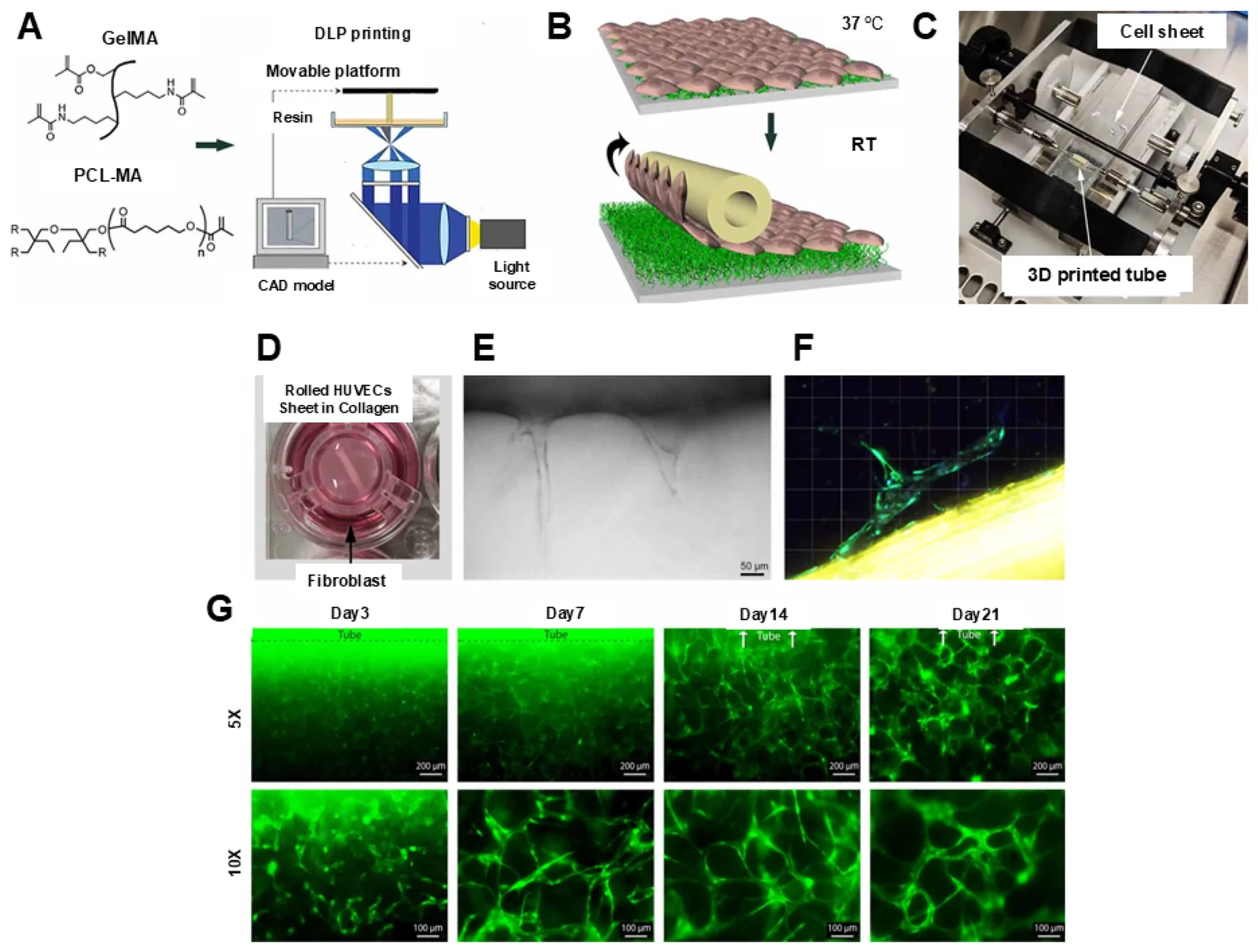
(**A**) Schematic illustration of the chemical structures of GelMA and PCL-MA used in the hybrid resin and the DLP 3D-printing process. (**B**) Detachment of a HUVEC cell sheet from a thermoresponsive culture surface and subsequently rolling into a tubular construct. (**C**) Photograph of the custom-designed device used for controlled cell-sheet rolling. (**D**) GFP-HUVEC-lined tubular scaffold embedded within a collagen matrix in a membrane insert. (**E**) Brightfield and (**F**) two-photon fluorescence microscopy images showing HUVEC sprouting from the rolled sheet (GFP, green; Hoechst, blue). (**G**) Representative fluorescence images of collagen gels containing both the tubular core and dispersed GFP-HUVECs (green), demonstrating the formation of vascular-like networks surrounding the central vessel-mimicking tube [10].

Tubular cell-sheet-based vascular grafts have been developed by wrapping multilayered cell sheets into vascular conduits, resulting in endothelialized constructs with organized smooth muscle cell layers after in vivo maturation [78]. Recent advances combine CSE with other biofabrication techniques and perfusion culture to achieve greater structural precision across large volumes and longer-term perfusion. Elomaa et al. combined a hybrid GelMA/PCL-MA 3D-printed tubular scaffold, with a HUVEC cell sheet. As depicted in Figure 3, after embedding into collagen hydrogel and coculturing with dermal fibroblasts, the construct elicited angiogenic sprouting and microvascular network development, which continued for 3 weeks [10]. The system formed dense, lumenized microvessels through HUVEC self-organization under dynamic perfusion, mediated by fibroblast-derived paracrine VEGF signaling. The construct achieved consistent viability and perfusion connection, representing one of the first demonstrations of an in vitro perfusable vascular bed fabricated by CSE around a 3D-printed perfusable vessel.

Combined CSE and electrospun fiber support for CSE systems enable more precise optimization of lumen geometry without sacrificing the biologically active sheet interface. Such integration thus overcomes diffusion limitations inherent to purely scaffold-free stacks, positioning CSE as a complementary bridge between decellularized matrices and synthetic scaffolds [79].

CSE in general offers a robust, biologically consistent approach to the creation of vascularized tissues. Dynamic perfusion, stem cell coculture, and improved manufacturing can produce multifaceted, perfusable vascular constructs that simulate native vessel physiology with significant translational potential for regenerative therapies. Nevertheless, technical bottlenecks include scaling-up of multilayer sheets, integration of vascular and parenchymal cell lineages, automating of sheet fabrication, and processes for safe handling and transfer of fragile sheets, e.g., thermoresponsive hydrogel-based robotic manipulation, to minimize mechanical stress and maintain viability [80]. Future directions for cell-sheet-based vascular engineering include incorporating microfluidic perfusion networks into stacked sheets to maintain continuous nutrient transport, as well as bioprinted hydrogel interlayers to improve adhesion between sheets and to enable directed endothelial migration. Allogeneic or stem-cell-derived cell-sheet banks also present a practical solution for future off-the-shelf and clinically scalable vascular grafts. The applications of electrospinning, decellularized matrices and cell-sheet engineering in vascularization are summarized in Table 1.

**Table 1:** Application of electrospinning, decellularized matrices and cell-sheet engineering on vascularization.

Strategies	Biomaterials	Properties	Cell Types	References
Electrospun scaffold	PCL/fibrin	Increased host microvessel density and reduced calcification, supporting enhanced cell infiltration and proliferation; neoarterial regeneration with ECM deposition and rapid endothelialization.	Cell-free	[81]
Electrospun scaffold	CS-g-PCL	Enhanced early angiogenesis and vascular ingrowth with improved biocompatibility in a rat femur chamber model.	Cell-free	[82]
Electrospun scaffold	PCL/ECM	Enhanced host endothelial and smooth muscle cell infiltration with elevated FLK-1, ICAM-1, and α-SMA expression at 2–4 weeks.	Cell-free	[83]
Decellularized matrices	Kidney dECM hydrogel	Promoted vascularized and more mature hPSC-derived kidney organoids in vitro and maintained vascular integrity after transplantation in mice.	hPSCs	[84]
Decellularized matrices	Decellularized dental pulp matrix	Retained native vasculature and angiogenic factors (VEGF-A, FGF-2) and, when seeded with hDPSCs, significantly enhanced angiogenic marker expression in vitro and vascularization in vivo.	hDPSCs	[85]
Decellularized matrices	Silk fibroin/decellularized vascular matrix gel	SF/DVMG hybrid scaffolds improved mechanical stability and degradation resistance while significantly enhancing endothelialization and vascular loop formation in vivo.	HUVECs	[86]
Cell-sheet engineering	Double-cell-sheet scaffold	Osteogenic–endothelial sheets enhanced mineralization and vascularization in vivo, with the osteogenic-over-endothelial configuration showing the best outcomes.	rADSCs	[87]
Cell-sheet engineering	Magnetic ASC and HUVEC cell sheets	Stratified heterotypic magnetic cell sheets (ASCs/HUVECs/ASCs) enhanced osteogenesis and angiogenesis, preserving human vascular structures and promoting vessel recruitment in the chick CAM model.	HUVECs and hADSCs	[88]
Cell-sheet engineering	Hypoxia-conditioned SVF-derived angiogenic cell sheets	Spontaneously formed capillary-like structures in vitro and significantly improved blood flow recovery in a mouse hind limb ischemia model.	SVF	[89]

CS = chitosan; PCL = polycaprolactone; ECM = extracellular matrix; dECM = decellularized ECM; SF = silk fibroin; DVMG = decellularized vessel matrix gel; hADSCs = human adipose-derived stem cells; rADSCs = rabbit adipose-derived stem cells; SVF = stromal vascular fraction; rBMSCs = rat bone marrow mesenchymal stem cells; hPSCs = human pluripotent stem cells; hDPSCs = human dental pulp stem cells; HUVECs = human umbilical vein endothelial cells.

### 4.4. 3D Printing and Sacrificial-Template Biofabrication

Three-dimensional (3D) printing is an attractive strategy for engineering complex vascularized tissues by enabling precise programmable deposition of biomaterials, cells, and biochemical signals. Compared to more conventional scaffold fabrication methods, 3D printing can produce hierarchical vascular architectures ranging from lumenized conduits and branching vascular trees to multicellular vessel walls, creating structures approaching the structural and functional complexity of native vasculature [90]. Advances in extrusion-based, inkjet-based, stereolithographic (SLA), digital light processing (DLP), and coaxial bioprinting technologies have extended the range of vessel sizes (17 μm^–1^ mm), and enhanced wall composition and mechanical properties (Table 2) [91,92].

Extrusion-based bioprinting continues to play a leading role in vascular applications because of its ability to print complex, cellularized viscous bioinks. This method enables printing of endothelial cells (ECs), smooth muscle cells (SMCs), fibroblasts, and support matrices to generate multilayered conduits that replicate tunica intima-, media-, and adventitia-like structures [93]. Inkjet bioprinting is superior for microscale patterning, although limited by cell density and bioink viscosity. Laser-assisted bioprinting enables high-precision patterning of single-cells. Recently, SLA and DLP bioprinting have enabled microscale fabrication of branched, perfusable networks in photocurable bioinks with high structural fidelity, including capillary-scale features on the order of a few micrometers [94].

**Figure 4:**
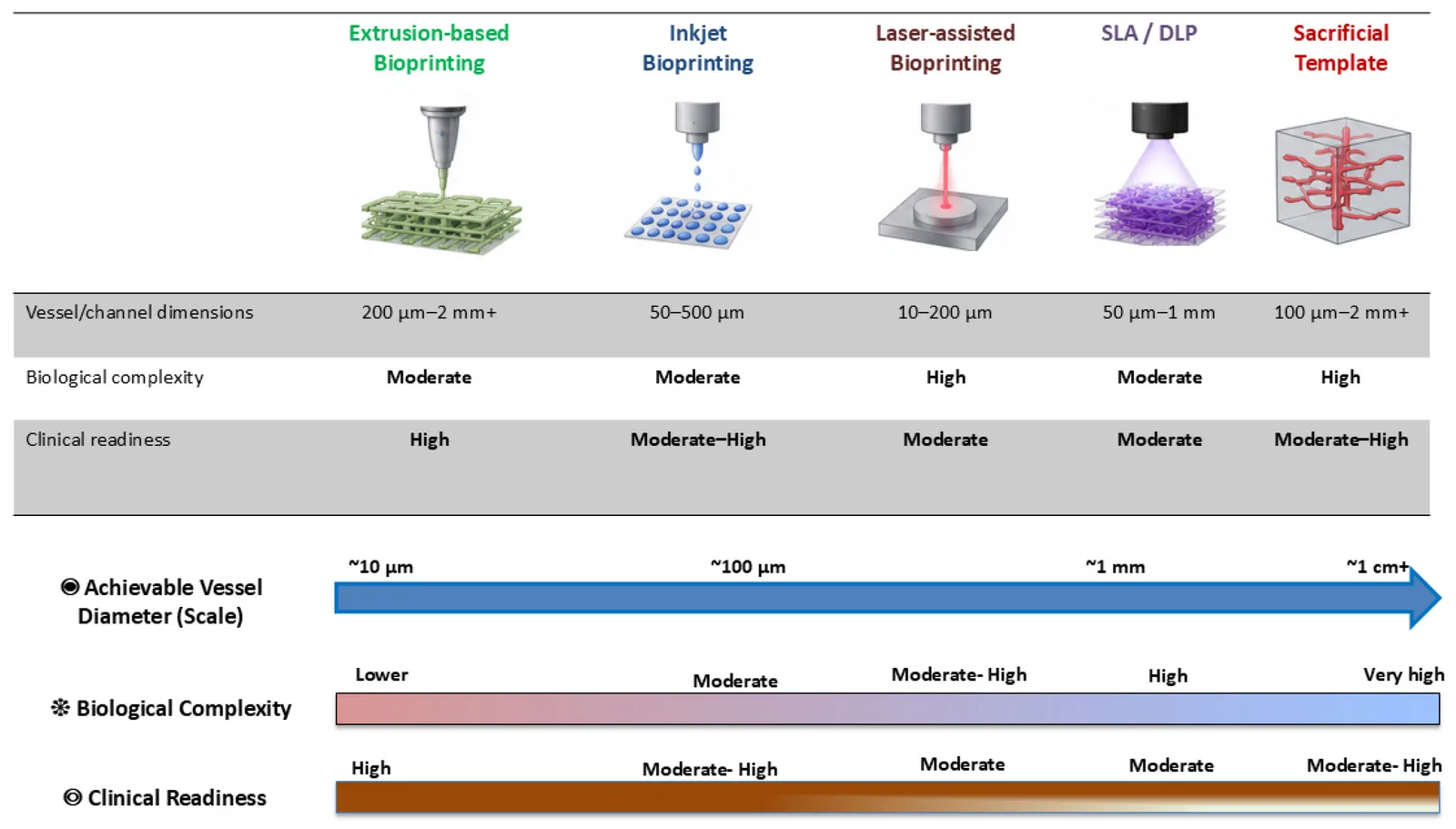
Relationships among major vascular fabrication strategies, achievable vessel or channel dimensions, biological complexity, and translational readiness. Hybrid approaches may combine complementary strengths; no single method currently reproduces the full dimensional and biological complexity of the native vascular tree.

The selection of a printing method depends on the required vessel size, material properties, and cellular complexity. Traditional extrusion-based printing is compatible with a broad range of viscous and cell-laden bioinks but suffers from lower spatial resolution. Inkjet printing provides finer microscale deposition but is limited to low-viscosity bioinks and lower cell densities. Laser-assisted printing enables precise, nozzle-free cell placement, although equipment complexity and scale-up remain limitations. SLA and DLP provide high geometric resolution but require photocrosslinkable materials and careful control of light exposure. Sacrificial-template approaches are particularly useful for forming perfusable channels within thick cell-laden matrices but require processing steps for template removal and subsequent cellularization of endothelial lining. Thus, no single printing method currently provides the resolution, material flexibility, biological complexity, and scalability needed to reproduce the complete vascular hierarchy (Figure 4) [95,96].

**Table 2:** Quantitative comparison of major 3D biofabrication strategies.

Technique	Representative Feature Resolution	Reported Vessel/Channel Scale	Material Compatibility	Biological/Technical Considerations	Major Advantages/Limitations	References
Extrusion-based	~200 µm	~200 µm to mm scale	High- and low-viscosity; cell-free, cell-laden, or cell-only inks	Broad material/cell compatibility; nozzle shear and lower resolution can limit capillary-scale printing	High scalability for larger conduits; limited direct capillary fabrication	[90,96,97,98,99]
Inkjet	~10–80 µm	~200 µm demonstrated	Primarily low-viscosity inks	Rapid microscale deposition; limited cell density and viscosity	Useful for precise patterning; less suited to thick mechanically robust constructs	[96,100]
Laser-assisted	~50–70 µm	Cell-width features (~10 µm) reported	Cell suspensions; low-viscosity formulations	Nozzle-free and precise cell placement; complex equipment and scale-up	Promising for microvascular patterning; limited large-scale manufacturing	[96,101]
SLA	~50–150 µm	Sub-mm to mm patterned or hollow structures	Photocrosslinkable bioinks/resins	High geometric fidelity; material selection and light exposure constrain cell-laden use	Promising where reproducibility and geometry are priorities	[93,94,95]
DLP	~10–50 µm	Features down to tens of µm; larger perfusable channels also feasible	Photocrosslinkable bioinks/resins	High resolution and rapid layer exposure; photoinitiator/light dose affect viability	Strong potential for reproducible high-resolution fabrication	[102,103]
Sacrificial template	~100–500 µm (typical)	~100–500 µm and larger perfusable channels	Gelatin, alginate, agarose, Pluronic F127, sugar-based inks	Enables perfusable channels in thick matrices; removal and endothelialization add steps	Strong potential for thick vascularized tissues and multiscale networks	[15,104,105,106,107,108,109]

3D printing facilitates the incorporation of angiogenic growth factors (e.g., VEGF, PDGF-BB, bFGF), exosomes, nitric oxide donors, and ECM-derived peptides within defined regions of the conduit [110]. This spatial patterning of biochemical cues promotes endothelial sprouting and directs mural cell migration, which facilitates rapid inosculation. Dynamic perfusion culture is still required to activate arterial mechanobiological responses to drive endothelial alignment, barrier formation, NO production, and matrix remodeling for functional maturation [111].

Extrusion-printing endothelial and stromal cells suspended in GelMA–fibrin composite bioinks enable self-assembly into lumenized microvascular networks that quickly inosculate into host vasculature following implantation [12,90,98,99,104,105]. 3D printing can also form constructs with growth-factor-releasing microspheres, which are effective in accelerating angiogenesis, innervation, and re-epithelialization of skin defects [106,110].

High-resolution 3D printing has enabled the fabrication of vascular constructs with controlled geometries and biologically relevant architectures. DLP-fabricated poly(propylene fumarate) (PPF) vascular grafts maintained patency for up to 6 months following implantation in a mouse venous model while coaxial bioprinting has enabled the fabrication of perfusable, multicellular vascular constructs containing endothelial and smooth muscle cells. Together, these studies demonstrate the potential of 3D printing to generate vascular conduits that combine geometric control with biologically relevant cellular organization and functional performance.

#### Sacrificial-Template 3D Printing for Perfusable Microvascular Networks

Despite the efficiency of traditional 3D printing in fabricating macro- and meso-scale vessels, it is challenging to reproducibly fabricate capillary-sized (<50 μm) and highly tortuous networks [112]. Printing a sacrificial material into a bulk hydrogel matrix creates perfusable channels after removal [113].

Sacrificial inks that produce perfusable channels can be categorized by the removal mechanism: thermal, ionic, enzymatic, solvent-based, or chemical. Hydrogel-based sacrificial inks, such as gelatin, alginate, and agarose, are still widely used [114]. The potential of tunable degradation (1–17 days) and controlled release of angiogenic factors, enabled by photo-crosslinked gelatin, has only recently been explored [114]. Protein–sugar composite inks, such as gelatin–sucrose matrices (GSM), result in increased mechanical stability and cytocompatibility, enabling perfusable vascular channels in human-scale constructs that maintain cell viability in volumetric tissue with minimal necrotic core formation [15]. Thermoresponsive polymers, like Pluronic F127 (PF-127), exhibit high print fidelity and reversible micellization, allowing for more sophisticated structures, including triple-coaxial prints in which PF-127 cores, collagen I (HUVEC-laden), and alginate (SMC-laden) layers create complex, perfusable vessel analogs possessing orderly endothelium and smooth muscle compartment arrangements [95,104]. PF-127 sacrificial channels integrated in fibrin constructs have provided perfusion within glioblastoma microenvironments in tumor models [115]. Finally, the sugar-glass template from sucrose offers a rigid structure, high resolution, and a rapid dissolution phase to obtain clean microchannels with high endothelial seeding efficiency [105,106]. Sacrificial networks result in endothelialized microchannels of ~500 μm or lower, approaching physiologic capillary scales, smaller than those achieved with current direct-printing techniques [109]. For example, embedded 3D printing with sacrificial ink in the SWIFT (sacrificial writing into functional tissue) approach fabricates perfusable, endothelial-lined multiscale vascular channels, producing a cardiac tissue construct that fused and exhibited synchronous beating over a 7-day period [107].

Sacrificial-template bioprinting requires careful configuration of material and fabrication parameters to meet biological needs. The selection of sacrificial material and its removal method must be compatible with the mechanical integrity of the hydrogel matrix and support cell viability [109]. In practice, thermoreversible, sugar–protein inks are favored when gentle, in situ dissolution is required to maintain extracellular matrix structure and protect embedded cells [116]. Finally, combining sacrificial networks with embedded bioprinting or support-bath bioprinting processes facilitates the fabrication of highly freeform, multiscale, vascular structures that more faithfully mimic native vascular networks [95]. The ongoing development of sacrificial chemistries and high-resolution printing is likely to advance the role of sacrificial-template bioprinting in the development of clinically relevant, vascularized tissues and organ constructs.

### 4.5. Microvasculature-on-a-Chip

Microvasculature-on-a-chip (MOC) and organ-on-a-chip (OOC) platforms provide valuable systems to study vascular physiology, disease mechanisms, and drug responses using biomolecules and living cells [117]. Compared to standard 2D culture or static 3D hydrogels, these microengineered systems incorporate microfluidics, multicellular co-culture, tissue-specific extracellular matrices (ECM), and biomechanical signals to simulate the morphology and function of human microvessels (Table 3) [3].

OOC-based systems, in the form of microvascular OOC models, typically follow design guidelines based on intrinsic study conditions observed in microcirculation in vivo, which consist of hierarchical branching, diffusion-limited oxygen transport, low-Reynolds-number (Re) laminar flow, and dynamic remodeling [118]. The control of microscale flow of oxygen and solutes through the membrane, interactions between endothelial and pericyte cells, and the 3D structure of the ECM represent capillary-level physiology with greater fidelity than a standard culture protocol [35].

The range of fabrication methods for soft lithography, viscous finger patterning, sacrificial molding, and hydrogel templating increases the accuracy of lumen geometry and supports multicellular arrangements, endothelial barrier function, ECM composition, perivascular support cells, active perfusion, and mechanotransduction required to represent human responses for disease modeling and pharmacological studies [119,120]. Microfluidic devices fabricated from PDMS, thermoplastics, or via hydrogel channel molding are the most utilized platforms for constructing microvasculature-on-chip systems [11]. Such devices generally include 10–150 μm perfusable microchannels that are lined with endothelial cells and subjected to a high degree of controllable shear stress (∼1–10 dyn cm^−2^) to model essential vascular functions such as barrier regulation, leukocyte adhesion, thrombosis, and mechanotransduction. Nevertheless, traditional microfluidic channels at this time are unable to recapitulate the tortuous architecture, dynamic sprouting behavior, and structural heterogeneity present in in vivo microvascular systems [11].

Microphysiological models embed endothelial cells and pericytes within collagen, fibrin, or Matrigel matrices to study sprouting angiogenesis, intussusceptive angiogenesis, and inosculation. Such systems closely replicate developmental and pathological angiogenesis and allow for modulating gradients, oxygen tension, and inflammatory activation studies [121,122,123]. These models also more precisely reproduce diffusion-dominated transport and capillary-scale vessel remodeling, and facilitate studies of permeability, tumor angiogenesis, and vessel–matrix interactions [124].

Microvascular OOC systems can integrate patterned microfluidic channels with self-maintained microvasculature for inosculation between engineered and native-like vessels. These hybrid architectures maintain dynamic flow, angiogenic sprouting from parent channels, endothelial barrier function, and immune cell trafficking. Consequently, vascularized OOC technologies are promising candidates for testing drug permeability and toxicity, and overcoming the inherent limitations of animal models [11]. They are therefore useful to model neurovascular events, especially blood–brain barrier and stroke pathophysiology, to screen for angiogenesis and anti-angiogenic therapy, and to analyze immune–vascular interactions, including leukocyte recruitment during flow [125]. Incorporating patient-derived iPSCs can increase physiological relevance and enable personalized drug-response profiling [126].

Advanced Vessel-on-a-Chip (VoC) chips, with endothelial cells, pericytes, and astrocytes, provide barrier integrity more than two orders of magnitude greater than that of endothelial-only systems, such that neuroinflammation and neurotoxicity can be quantified in a highly predictive manner [127]. Microfluidic models of tumor–vascular interaction can be used to image intravasation and extravasation in real time, demonstrating shear-dependent endothelial activation as tumors progresses toward metastasis [128]. Diabetic microangiopathy chips have been developed to reflect significant vascular defects such as basement membrane thickening, inflammatory activation, and reduced nitric oxide bioavailability under conditions of increased glucose and cytokine production [129]. Heart-on-a-Chip models include metabolic coupling with contracting cardiomyocytes and flow-responsive regulation of oxidative stress can provide valuable insights into ischemia, cardiotoxicity, and microvascular dysfunction [130,131].

Taken together, microvascular OOC technologies can monitor structural, biochemical, and mechanobiological characteristics of human microcirculation that are frequently inaccessible in animal models or static cultures. As these advances continue to develop, integration with stem-cell-derived organoids, machine-learning-facilitated analytics, multi-organ interconnecting platforms, and fully synthetic ECM analogs will further increase physiological fidelity and accelerate clinical translation.

**Table 3:** Representative applications of 3D-printed scaffolds and microvascular-on-a-chip platforms for vascularization.

Strategies	Biomaterials	Properties	Cell Type	References
3D Printing	GelMA and PEGDA	SLA/PLP/FDM-fabricated bone constructs with sacrificial vascular channels promoted MSC-driven osteogenesis and HUVEC-mediated capillary sprouting under perfusion culture.	hBM-MSCs; HUVECs	[132]
3D Printing	PCL	Deferoxamine-releasing 3D-printed scaffolds enhanced angiogenesis and osteogenesis in vitro and promoted vascular ingrowth and bone regeneration in a rat critical-sized bone defect model.	rBM-MSC; HUVECs	[133]
3D Printing	TCP/PLGA	Dual-delivery 3D-printed scaffolds enabled rapid angiogenesis and enhanced bone formation via sequential release of angiogenic and osteogenic peptides in a rat bone defect model.	rBM-MSC; Rat endothelial cells	[134]
3D Printing	Silk–hydroxyapatite	3D-printed scaffolds functionalized with BMP-2, VEGF, and NGF synergistically enhanced osteogenic differentiation and supported endothelial and neural cell activity in vitro.	hBM-MSC; HUVECs and hiNSCs	[135]
microvasculature-on-a-chip	PDMS	Nintedanib impaired microvascular network formation by increasing vessel permeability and reducing microvessel density and diameter in a perfusable in vitro lung microvasculature model.	HUVECs; hNL-FBs	[136]
microvasculature-on-a-chip	PDMS	Direct inclusion of fibroblasts was essential for forming thin, interconnected, and perfusable microvascular networks, while soluble cues alone produced nonphysiological vascular morphologies in vitro.	HUVECs; hNL-FBs	[137]
microvasculature-on-a-chip	PDMS	Lung fibroblasts most effectively supported endothelial morphogenesis, yielding highly interconnected, perfusable microvascular networks in a microfluidic in vitro model.	HUVECs; mBM-MSC; mLFs	[138]

GelMA = methacrylated gelatin; PEGDA = polyethylene glycol diacrylate; SLA = stereolithography; PLP = Projection Light Processing; FDM = fused deposition modeling; TCP = tricalcium phosphate; PLGA = poly(lactic-co-glycolic acid); PDMS = polydimethylsiloxane; HUVECs = Human Umbilical Vein Endothelial Cells; hBM-MSCs = human bone marrow-derived mesenchymal stem cells; rBM-MSCs = rat bone marrow-derived mesenchymal stem cells; hiNSCs = human Induced Neural Stem Cells; hNL-FBs = human normal lung fibroblasts; mBM-MSCs = mouse bone marrow-derived mesenchymal stem cells; mLFs = mouse lung fibroblasts.

## 5. Cell Sources for Vascularization

Engineering vascular conduits with long-term patency and physiological function is primarily dependent on a stable, non-thrombogenic endothelium. Endothelial cells are responsible for hemostasis, barrier permeability, leukocyte trafficking, and vasomotor tone. A dysfunctional endothelium promotes platelet adhesion, fibrin accumulation, and inflammatory activation, ultimately leading to graft occlusion (reviewed in [139]). Therefore, the choice of endothelial cell source, the support of mural cells, and preconditioning regimens are significant design considerations for vascular graft specification.

### 5.1. Endothelial Cell Sources

#### 5.1.1. Primary Endothelial Cells

Primary human umbilical vein endothelial cells (HUVECs) and human dermal microvascular endothelial cells (HDMVECs) are among the most commonly used, as they are readily obtained, exhibit robust angiogenic activity, and are highly reproducible in vitro. Nevertheless, these terminally differentiated cells with low proliferative potential for scaling up and high donor-to-donor variability have limited potential for clinical translation.

#### 5.1.2. Endothelial Progenitor Cells (EPCs)

Endothelial progenitor cells (EPCs), including late-outgrowth endothelial colony-forming cells (ECFCs), represent a progenitor-like endothelial lineage with high clonogenic and vasculogenic potential [140]. EPCs can be isolated from peripheral or cord blood and form stable, perfusable vessels in vivo [141]. But donor heterogeneity and diminished EPC function in metabolic or cardiovascular patients are key limitations that must be further investigated [142]. Emerging studies indicate that cord blood-derived ECFCs are more attractive for regenerative use. The greater proliferation and network-formation potential and lower immunogenicity than adult endothelial sources support the possibility that ECFCs could function as a unique source of endothelium for prevascularized constructs [143].

#### 5.1.3. Pluripotent Stem Cell–Derived Endothelial Cells

Endothelial cells generated from induced pluripotent stem cells (iPSCs) can be efficiently differentiated into endothelial cells, which recapitulate central endothelial activities, such as nitric oxide generation, LDL uptake, or endothelium-mediated endothelial tube development. Their autologous nature reduces the risk of immune rejection, but genetic stability, epigenetic memory, and risk of residual pluripotency have limited their clinical application [144]. Human venous endothelial cells (iVECs) derived from iPSCs can mimic vascular malformations and have been used to screen for drugs, showing functional vascular networks capable of being employed to identify therapeutics that rescue disease phenotypes in vitro and in vivo [145]. A CD157^+^ subclass of iPSC-derived endothelial cells, with notably favorable angiogenic potential, indicates that endothelial stem-like cells can be present in iPSC-derived EC populations [145,146].

Immunogenicity is a key issue for translation. Relative to primary HUVECs, human iPSC-derived ECs have low expression of immunologically relevant surface molecules, which suppresses the activity of immune cells and supports their use in allogeneic or hypoimmunogenic graft strategies [147]. Taken together, these studies position iPSC-derived ECs as a potential candidate for stand-alone vascular lining of engineered grafts, provided that genetic stability and residual pluripotency are adequately addressed.

### 5.2. Supporting Cells

Endothelial monolayers alone are insufficient to produce and sustain durable or physiologically stable vascular systems. Mural cells in native vessels play a crucial role in mechanical reinforcement, regulation of barrier integrity, modulation of vasomotor tone, and regulation of vessel morphogenesis. Pericytes, which envelop capillaries and post-capillary venules, contribute to basement membrane deposition, vessel stabilization, and endothelial permeability. Pericytes protect from mechanical and inflammatory stress, facilitate long-term structural functionality, and serve as a critical cellular component for engineered microvasculature stabilization [148]. At a mechanistic level, mural cell-derived matrix-bound VEGF is indispensable for endothelial morphogenesis and lumen formation, a process that cannot be mimicked with soluble VEGF alone [149]. Mural cells also interact with stabilizing pathways (PDGFRβ, TGF-β, and Ang1–Tie2 signaling) to induce endothelial quiescence and inhibit aberrant sprouting. In vivo, engineered pericytes or VSMC-coated constructs result in superior perfusion, faster inosculation, and long-term patency, compared with endothelial-only grafts, highlighting the critical role of mural cells in functional vessel integration [150].

### 5.3. Vascular Smooth Muscle Cells (VSMCs)

VSMCs are the primary contractile cells in the walls of arterioles and arteries, providing vascular compliance and regulating vasoreactivity. The development of conduits that replicate biomechanical behavior and active vasomotor responses of arteries require primary or iPSC-derived VSMCs [151]. iPSC-derived SMCs improve endothelial network development and enhance vessel stability when embedded in fibrin- or collagen-based hydrogels, demonstrating structural and paracrine support for engineered vessels [152].

### 5.4. Mesenchymal Stem/Stromal Cells (MSCs)

MSCs are a diverse stromal component, contributing paracrine pro-angiogenic signals (e.g., VEGF, PDGF-BB) that support differentiation toward pericyte- or smooth muscle-like phenotypes [153]. Recent studies have evaluated MSCs as a stand-alone stromal support for vascularization. For example, human mesenchymal stem cells enhance the viability of HUVECs, promote the growth of lumenized vascular sprouts with branch points, and bind endothelium (perivascular repair) under flow [154]. The source of MSC tissue (i.e., bone marrow versus adipose) significantly affects the maturation and function of the microvascular network, underscoring the importance of MSC origin in the design of prevascularized constructs [155].

### 5.5. Coculture Strategies

The feasibility and durability of vascularization generally require coculture of endothelial and mural lineages. While EC-to-mural cell ratios ranging from 1:1 to 3:2 are common, the optimal ratio depends on the cell types and the matrix environment [148]. In 3D fibrin constructs, coculture of endothelial cells and stromal/MSC populations support simultaneous osteogenesis and vasculogenesis, and prevascularized networks persist and renew over time, emphasizing the combined effect of stromal and endothelial compartments [156]. Coculture of endothelial lineage cells with MSCs, fibroblasts, and perivascular cells consistently results in a more developed and perfusable microvascular networks than monocultures of single lineages [12].

Cocultured cells are bidirectionally beneficial. EC cocultured with MSC in 3D endochondral ossification organoids promote better vascularization and subsequent tissue (bone) regeneration than using MSC-only constructs [157]. MSCs provided trophic and protective support to endothelial colony-forming cells (ECFCs) in cellular spheroids significantly improved in vivo survival and angiogenic engraftment as compared with ECFCs alone [158].

In addition to cellular organization, medium composition and culture conditions play a key role in determining outcomes. A recent study defined minimum medium conditions under which HUVECs with stromal cells in 3D hydrogel coculture can self-integrate and lumenize into stable vascular networks. Optimized and efficient biochemical environment can reduce dependence on high levels of exogenous growth factors like VEGF [159].

Mechanical and structural conditioning also enhances vascular graft maturation. Cocultured endothelial and smooth muscle cells under physiologically relevant mechanical constraints improved the spontaneous orthogonal alignment of the two cell types, mimicking native vessel morphology [160]. This alignment likely enhances the mechanical properties and long-term structural integrity of engineered vessels. These findings are translationally relevant: for example, combination cell-based prevascularization (ECs with stromal or mural cells) is emerging as a cornerstone in strategies for improving graft acceptance and integration after implantation in vascularized skin substitutes [160].

Taken together, these studies support the potential utility of multiple endothelial and mural cell lineages: primary ECs, EPCs/ECFCs, iPSC-derived ECs, pericytes, VSMCs, and MSCs, as important factors for vascularization strategies. The incorporation of these cell types into well-designed coculture systems is a central focus of ongoing innovation in developing perfusable, durable, and biologically responsive vascular networks.

## 6. Future Directions

Future progress in vascular biofabrication will require significant advances in biological performance and manufacturing. Scalable production remains challenging for constructs that combine multiple cell populations and various biomaterials over vascular length scales. Functional performance assays will be needed to standardize identity, purity, sterility potency, and reduce batch-to-batch variability. Regulatory strategies must also account for the combination nature of these products, which incorporate living cells, degradable biomaterials, and bioactive factors in device-based manufacturing processes.

Artificial intelligence and computational approaches may assist with optimization of printing parameters, vascular geometry, bioink composition, and perfusion conditions using experimental, imaging, and process data. Smart biomaterials responsive to mechanical, biochemical, or cellular cues may enable controllable degradation, growth-factor presentation and delivery, endothelialization, and remodeling. Clinical translation will ultimately require these advances to be combined with scalable cell sources, automated and closed manufacturing workflows, long-term in vivo validation, reliable host integration, and clearly defined release criteria. Future studies should prioritize an adaptable “systems approach” that can meet biological performance and manufacturing-regulatory requirements, rather than focusing primarily on optimizing individual specifications, e.g., printing resolution.

## 7. Conclusions

The engineering of functional multiscale vascular networks remains a critical and complex challenge for tissue engineering and regenerative medicine. Considerable progress has been made through diverse approaches, such as electrospun scaffolds, decellularized matrices, cell-sheet engineering, advanced three-dimensional bioprinting, and sacrificial-template fabrication, each addressing specific aspects of vascular structure and function. Achieving successful vascularization requires integrating hierarchical architecture, stable endothelialization, mural cell support, and physiologically relevant mechanical and biochemical cues. Concurrently, advances in cell sourcing, particularly the development of endothelial progenitors and induced pluripotent stem cell (iPSC)-derived vascular lineages, together with optimized coculture and preconditioning strategies, have significantly enhanced vessel stability, perfusion, and host integration. Microvascular and organ-on-a-chip platforms have further advanced the field by enabling high-fidelity modeling of human vascular physiology and disease, providing powerful tools for mechanistic studies and therapeutic discovery. The synthesis of biomaterial science, stem cell technology, and biofabrication provides new opportunities to develop vascular grafts that can remodel, grow, and integrate functionally into endogenous tissues [161].

However, major barriers to clinical translation include: (1) reproducible and scalable manufacturing, which requires reliable fabrication and maturation of capillary-scale networks; (2) enhancing performance after implantation which requires immune compatibility, reproducible host anastomosis or inosculation, preservation of long-term patency after remodeling, and preservation of tissue-specific endothelial phenotypes; and (3) establishing and meeting regulatory standards which requires standardizing quality-control assays and developing regulatory pathways for complex cell–biomaterial combination products. Advances in vascular biology, biomaterials science, stem cell engineering, biofabrication, and translational manufacturing must be carefully coordinated to produce clinically viable constructs capable of sustaining functional perfusion in vivo.

## Data Availability

No new data were created or analyzed in this study. Data sharing is not applicable to this article.
